# Parthenolide Suppresses T Helper 17 and Alleviates Experimental Autoimmune Encephalomyelitis

**DOI:** 10.3389/fimmu.2022.856694

**Published:** 2022-04-20

**Authors:** Zhihui Zhang, Kai Zhang, Mi Zhang, Xiaomin Zhang, Rongxin Zhang

**Affiliations:** ^1^ Tianjin Key Laboratory of Retinal Functions and Diseases, Tianjin Branch of National Clinical Research Center for Ocular Disease, Eye Institute and School of Optometry, Tianjin Medical University Eye Hospital, Tianjin, China; ^2^ Department of Chemistry and Biology, Ryerson University, Toronto, ON, Canada; ^3^ Guangdong Province Key Laboratory for Biotechnology Drug Candidates, Institute of Basic Medical Sciences, Guangdong Pharmaceutical University, Guangzhou, China; ^4^ Department of Biotechnology, School of Life Sciences and Biopharmaceutics, Guangdong Pharmaceutical University, Guangzhou, China

**Keywords:** multiple sclerosis (MS) disease, T helper 17 cell (Th17 cell), parthenolide (PTL), nuclear factor kappa B (NF-κB), experimental autoimmune encephalomyelitis (EAE), retinoid-related orphan receptor-γt (RORγt)

## Abstract

T helper (Th) cells play crucial roles in inflammation and adaptive immune system. Importantly, Th17 cells, a major pathogenic Th cell subset, are involved in the pathogenesis of multiple sclerosis (MS) and its classical animal modal experimental autoimmune encephalomyelitis (EAE). Previous studies have shown that parthenolide (PTL), a sesquiterpene lactone, possesses potent anti-cancer and anti-inflammatory activities. However, the immunosuppressive effect of PTL on the pathogenic Th17 cell and MS is unclear. In this study, we showed that PTL treatment could alleviate clinical symptoms by inhibiting inflammatory cell infiltration, reducing inflammation and demyelination of CNS. In addition, the mRNA expression of cytokines and inflammatory factors in CD4^+^ T cells, especially Th1 and Th17 cells, reduced in both CNS and peripheral immune tissue of EAE mice. Furthermore, PTL could inhibit the reactivation of MOG-specific T cells and the differentiation of naïve CD4^+^ T cells into Th17 cells *in vitro*. We also found that PTL inhibited nuclear factor kappa B (NF-κB) signaling and retinoid-related orphan receptor-γt (RORγt) in mouse Th17 cell and human Jurkat cell line. Taken together, our data demonstrated a critical immune-suppressive effect of PTL on autoimmune inflammation through regulating Th17 cells and the NF-κB/RORγt pathway.

## Introduction

Multiple sclerosis (MS) is a chronic autoimmune inflammatory demyelinating disease with progressive damage to axons in the central nervous system (CNS) (1) and is mainly triggered by the CD4^+^ T cells’ response to myelin antigens (2). Previous studies suggested that T helper (Th) 1 cell differentiation is crucial during the development and progression of MS (3–5). Abnormal activation of other T helper cells, such as Th2 and Treg cells, is also related to the occurrence of MS (6, 7). However, with the discovery of Th17 cells in 2005, more and more studies have shown that Th17 cells are involved in the development of autoimmune syndromes, such as MS (8–10). Th17 cells produce interleukin (IL)17 together with many other proinflammatory cytokines, including IL-17F, IL-22, IL-26, and granulocyte-macrophage colony-stimulating factor (GM-CSF), further inducing the expression of tumor necrosis factor α (TNFα), IL-6, granulocyte colony-stimulating factor (G-CSF), IL-1, and the chemokine ligand 1, causing tissue-specific inflammation (11).

Parthenolide (PTL) is a major sesquiterpene lactone, derived from the medicinal plant feverfew (*Tanacetum parthenium*), which belongs to a variety of natural products that have been used for their potential anti-inflammatory and anti-cancer properties (12–15). Studies have shown that PTL is a kind of nuclear factor kappa B (NF-κB) inhibitor that can inhibit the NF-κB pathway by directly binding to NF-κB subunits (15, 16). Different from other NF-κB inhibitors with antioxidant properties, PTL does not possess radical-scavenging activity (17). Besides, PTL has other biological abilities, such as inducing global DNA hypomethylation *via* specifically suppressing DNA methyltransferase 1 (DNMT1) activity *in vitro* and *in vivo* and showing high potency against leukemia (18, 19). Although PTL is mainly studied in cancer treatment (20, 21), current studies showed that PTL can affect Th17/Treg immune balance by regulating the metabolism of gut microbiota (22), alleviate peritoneal fibrosis by suppressing the TGF-β/Smad pathway (23), inhibit the initiation of experimental autoimmune neuritis, and alleviate collagen-induced arthritis. However, the exact effects of PTL on MS remains elusive.

The NF-κB signaling pathway plays important roles in immune and inflammatory diseases, such as MS, rheumatoid arthritis, and inflammatory enteritis (24, 25). The NF-κB family has five members including NF-κB1 (p105), NF-κB2 (p100), RelA (p65), RelB, and c-Rel. All of these members are present as either homo- or heterodimers in cells (26). In T cells, the major NF-κB subunits are p50/c-Rel or p50/p65 heterodimers, which could be activated by T-cell receptor (TCR) stimulation (27). T cells that are separated from c-Rel-knockout mice express lower levels of CD25 and fail to differentiate into functional effector T cells. In addition, c-Rel-knockout mice show the potential of resistance to experimental autoimmune encephalomyelitis (EAE) induced by myelin oligodendrocyte glycoprotein (MOG) (28). Moreover, RelA (p65) is crucial for producing IL-17 in γδT cells (29). Also, studies showed that NF-κB1 (p105/50) deficiency could significantly protect mice from EAE (30). Studies have demonstrated that a-Rel (p65) could combine to the Rorc gene and control the Th17 transcription factor ROR-γt, further affecting the differentiation of Th17 cells (31, 32).

In this study, we found that PTL could alleviate the clinical symptoms and reduce the infiltration of inflammatory cells in the CNS of EAE. We also explore the immunosuppressive role of PTL in the inflammatory response of Th1 and Th17 cells in the peripheral and central nervous system, as well as the potential regulatory mechanism.

## Materials and Methods

### Animals

C57BL/6 female adult mice (6–8 weeks) were purchased from Vital River (Beijing, China). The animals were housed in a pathogen-free standard cage, fed with normal mouse chow and water *ad libitum*, and maintained a 12-h on/off light cycle at the Experimental Animal Center of Tianjin Medical University eye hospital (Tianjin, China). The experiments were performed in accordance with the guidelines for animal care and were approved by the Animal Ethics Committee of Tianjin Medical University eye hospital (Tianjin, China).

### EAE induction

EAE was immunized as previously described (33). Briefly, 150 μg of Myelin oligodendrocyte glycoprotein (MOG)35-55 peptide (CL.Bio-Scientific CO., LTD, Xi’an, China) was emulsified in complete Freund’s adjuvant (Difco, Detroit, MI) containing 5 mg/ml of heat-killed *Mycobacterium tuberculosis* H37RA (Difco, Detroit, MI) and subcutaneously (s.c.) injected into both flanks of C57BL/6 mice (6–8 weeks) on day 0. On day 0 and day 2, 200 ng of pertussis toxin (List Biological Laboratories, Campbell, CA) in 200 μl of PBS was given to each mouse *via* tail vein. The peptide sequence is Met-Glu-Val-Gly-Trp-Tyr-Arg-Ser-Pro-Phe-Ser-Arg-Val-Val-His-Leu-Tyr-Arg-Asn-Gly-Lys and the purity is >95% (CL Bio-Scientific Co., Ltd., Xi’an, China).

### PTL Administration

PTL was purchased from Tianjin Shilan Technology company, purity >98%, and dissolved in DMSO (dimethyl sulfoxide; Sigma-Aldrich, Missouri, USA). For the treated group, PTL was intraperitoneally administered at 10 mg/kg of body weight three times per week from day 1 after EAE induction according to the previous reports (14, 22, 34). For the therapy group, mice that showed clinical symptoms on day 11 after induction were selected and randomly divided into two groups to receive PTL 10 mg/kg of body weight three times per week or DMSO (22).

### Clinical Scoring

Clinical scores of EAE were measured daily from day 0 to day 28. The EAE mice were weighed and examined daily, and disease symptoms were assessed using the following standard score system: 0, no obvious changes in motor functions; 1.0, limp tail; 2.0, limp tail and wobbly gait; 3.0, bilateral hind limb paralysis; 4.0, complete hind limb and partial fore limb paralysis and 5.0, death (35). This scale allows the assessment of subtle changes in neuronal pathology. The clinical score of 1.0 or above is used to calculate the incidence rate, and <0.5 is the recovery period. The clinical scoring was evaluated and calculated in a blinded manner.

### Histology

Mice were sacrificed at day 21 after immunization and the lumbar segment of spinal cords from EAE mice pretreated with PTL or DMSO were removed and fixed in 4% paraformaldehyde. Fixed spinal cords were embedded with paraffin and cut into 10-μm sections. Hematoxylin-eosin (H&E) and Luxol fast blue (Alfa Aesar, Ward Hill, USA) were used to examine the inflammatory infiltration and demyelination, respectively. The percentage of inflammatory infiltration and demyelination areas were measured by software ImageJ.

### Preparation of Single-Cell Suspensions From CNS

Animals were perfused with cold PBS. The brains and spinal cords were dissected and passed through a 70-μm strainer. Then, the cells were incubated in 2.5 mg/ml collagenase D for 60 min at 37°C. The cells were washed in RPMI 1640 medium, and mononuclear cells were isolated using a discontinuous Percoll gradient (Pharmacia).

### Flow Cytometry

Single-cell suspensions were separated from spleens, lymph nodes, and spinal cords of DMSO or PTL-treated EAE mice at onset and peak phase. For Th1, Th2, and Th17 cell staining, cells were cultured (in triplicate) in complete RPMI 1640 medium (containing 100 mM sodium pyruvate, 200 mM L-glutamine, 1 mg/ml penicillin/streptomycin, and 10% fetal bovine serum) and stimulated with 50 ng/ml PMA (phorbol 12-myristate 13-acetate; Enzo Life Sciences, Farmingdale, USA), 500 ng/ml ionomycin (Enzo Life Sciences, Farmingdale, USA), and 1 μg/ml Brefeldin A (Sigma-Aldrich, Missouri, USA) for 5 h. Cells were washed with PBS and stained with APC-conjugated rat anti-mouse CD4 (eBioscience, CA, USA). Next, the cells were fixed, permeabilized, and stained with PE-conjugated rat anti-mouse IL-17A (eBioscience, CA, USA), FITC-conjugated rat anti-mouse IFN-γ (eBioscience, CA, USA), and PE-conjugated rat anti-mouse IL-4 (eBioscience, CA, USA). Nonspecific staining was monitored with isotype antibody controls. For Treg cell staining, cells without stimulation were directly stained with cell surface markers including APC-conjugated rat anti-mouse CD4 (eBioscience, CA, USA) and FITC-conjugated rat anti-mouse CD25 (eBioscience, CA, USA). Next, the cells were fixed, permeabilized, and stained by PE-conjugated rat anti-mouse Foxp3 (eBioscience, CA, USA). BD FACSCelesta (BD, CA, USA) was used to measure the cells and FlowJo software (Tree star, Ashland, OR) was used to analyze the FACS data.

### Real-Time qPCR

The total mRNA was extracted from PTL or DMSO-treated mouse CNS, spleen, lymph node tissue, or differentiation Th17 cells by using TRIzol reagent (Invitrogen, CA, USA). Random hexamers and M-MLV reverse transcriptase (Invitrogen, Oregon, USA) were used for the conversion of mRNA to cDNA. The mRNA expression was measured by using SYBR green mix (Newbio industry, Beijing, China) and the ABI 7500 fast instrument (Applied Biosystems, Foster City, CA). The primer sequences of target genes were as follows:

IL-4 Forward: TCATCGGCATTTTGAACGAG
Reverse: TTTGGCACATCCATCTCCG
IL-6 Forward: AGCCAGAGTCCTTCAGAGAG
Reverse: GATGGTCTTGGTCCTTAGCC
IL-17a Forward: CTCCAGAAGGCCCTCAGACTAC
Reverse: AGCTTTCCCTCCGCATTGACACAG
IL-17f Forward: GAGGATAACACTGTGAGAGTTGAC
Reverse: GAGTTCATGGTGCTGTCTTCC
IL-22 Forward: CATGCAGGAGGTGGTACCTT
Reverse: CAGACGCAAGCATTTCTCAG
IFN-γ Forward: GCATTCATGAGTATTGCCAAGTTT
Reverse: GATTCCGGCAACAGCTGGT
TNF-α Forward: ACCCTCACACTCAGATCATC
Reverse: GAGTAGACAAGGTACAACCC
T-bet Forward: GCCAGGGAACCGCTTATATG
Reverse: GACGATCATCTGGGTCACATTGT
RORc Forward: AGTGTAATGTGGCCTACTCCT
Reverse: GCTGCTGTTGCAGTTGTTTCT
IL-21 Forward: GCCTCCTGATTAGACTTCGTCAC
Reverse: CAGGCAAAAGCTGCATGCTCAC
GM-CSF Forward: GGCCTTGGAAGCATGTAGAGG
Reverse: GGAGAACTCGTTAGAGACGACTT
GATA3 Forward: CCTCTGGAGGAGGAACGCTAAT
Reverse: GTTTCGGGTCTGGATGCCTTCT
Foxp3 Forward: CCTGGTTGTGAGAAGGTCTTCG
Reverse: TGCTCCAGAGACTGCACCACTT
IL-10 Forward: CGGGAAGACAATAACTGCACCC
Reverse: CGGTTAGCAGTATGTTGTCCAGC
GAPDH Forward: ACCACAGTCCATGCCATCAC
Reverse: TCCACCACCCTGTTGCTGTA

### T-Cell Proliferation and Cytokine Array

At day 21 after immunization, splenocytes (5 × 10^5^/well) were separated from PTL- or DMSO-treated EAE mice and cultured in 96-well plates with complete medium. Cells were treated without or with different doses of the MOG_35–55_ peptide at 37°C in 5% CO_2_ for 72 h. The proliferation rate was measured using the colorimetric Cell Proliferation ELISA BrdU Kit (Roche, Basel, Switzerland).

At day 21 after immunization, splenocytes (5 × 10^5^/well) were separated from EAE mice and cultured in 96-well plate with complete medium. Cells were activated with MOG_35–55_ peptide (20 μg/ml), cultured without or with different doses of PTL at 37°C in 5% CO_2_ for 72 h. The proliferation rate was measured using the colorimetric Cell Proliferation ELISA BrdU Kit (Roche, Basel, Switzerland).

*Ex vivo* T-cell recall assays were performed on day 21 after immunization. Splenocytes (5×10^5^) were cultured in 96-well with 100 µl of RPMI 1640 medium with or without 20 µg/ml of peptide MOG35–55 peptide or a non-relevant peptide (ovalbumin OVA323–339; Sigma-Aldrich, Missouri, USA). After 72 h, the supernatants were collected and the IFN-γ and IL-17A were measured by using ELISA kits (BioLegend, CA, USA) following the manufacturer’s instructions.

### T Helper Cell Differentiation

Total CD4^+^ naïve T cells were isolated from spleen and lymph nodes by using Magnisort™ Mouse CD4 naïve T cells Enrichment Kit (Invitrogen, CA, USA) according to the manufacturer’s instructions. Twenty-four-well plates were pre-coated with anti-mouse CD3 mAb (5µg/ml; BioLegend, CA, USA) and anti-mouse CD28 mAb (2 µg/ml; BioLegend, CA, USA) at 4°C overnight. For Th17 cell differentiation, 5×10^5^ CD4^+^ T cells were seeded in the 24-well plates and cultured at Th17 polarizing condition medium supplemented with IL-6 (20 ng/ml), TGF-b1 (2 ng/ml), anti-IL4 (10 µg/ml), and anti-IFN-γ (10 µg/ml) for 4 days. For Th1 cell differentiation, equal amount of CD4^+^ T cells were seeded in the 24-well plate and cultured at Th1 polarizing condition medium supplemented with IL-12 (20 ng/ml), anti-IL4 (10 µg/ml) for 4 days. For Treg cell differentiation, equal amounts of CD4^+^ T cells were seeded in the 24-well plates and cultured at Treg polarizing condition medium supplemented with TGF-b1(5 ng/ml) and IL-2 (2 ng/ml) for 4 days. During the differentiation, cells were treated with PTL (3 μM). After the differentiation, cells were collected and detected by FACS. All cytokines were purchased from R&D Systems and BD Biosciences. mRNA expression of Th1-, Treg-, and Th17-related cytokines, transcription factors, and surface receptors was measured by RT-qPCR.

### Western Blot

Western blotting was used to detect the expression of pIκBα, IκBα, c-Rel, p65, and ROR-γt protein in the Jurkat cells and Th17 cells. Naïve CD4^+^ T cells were cultured under Th17 polarizing conditions with PTL (0, 1, and 3 μM) for 3 days. Jurkat cells were treated with PTL (0, 1, and 3 μM) and incubated for 24 h. For total protein extraction, RIPA lysis buffer (Sigma-Aldrich, Missouri, USA) with protease and phosphatase inhibitor mixture (Sigma-Aldrich, Missouri, USA) was used for the whole-cell lysate. Nucleoprotein extract was followed by nuclear and cytoplasmic extraction reagents (Thermo Fisher, Waltham, USA). Protein concentration was determined by BCA protein assay kit (Sigma-Aldrich, Missouri, USA). Proteins were detected using various primary antibodies and antibody–antigen complexes were measured by the Chemiluminescent HRP Substrate (Millipore, MA, USA). Antibody for pIκBα, IκBα, c-Rel, and p65 were purchased from Cell Signaling. RORγt were purchased from Abcam. β-actin was purchased from Sungene.

### Statistical Analysis

Statistical significance was determined by unpaired Student**’**s ***t***-test, two-way analysis of variance (ANOVA), and Mann–Whitney ***U*** test. In all the statistical comparisons, when *p* value < 0.05, the difference was considered to be significant (*p < 0.05, **p < 0.01, ***p < 0.001). All the data were presented as the mean ± standard deviation (SD).

## Results

### PTL Ameliorated the Clinical Symptoms of Mice With EAE

**Figure 1:**
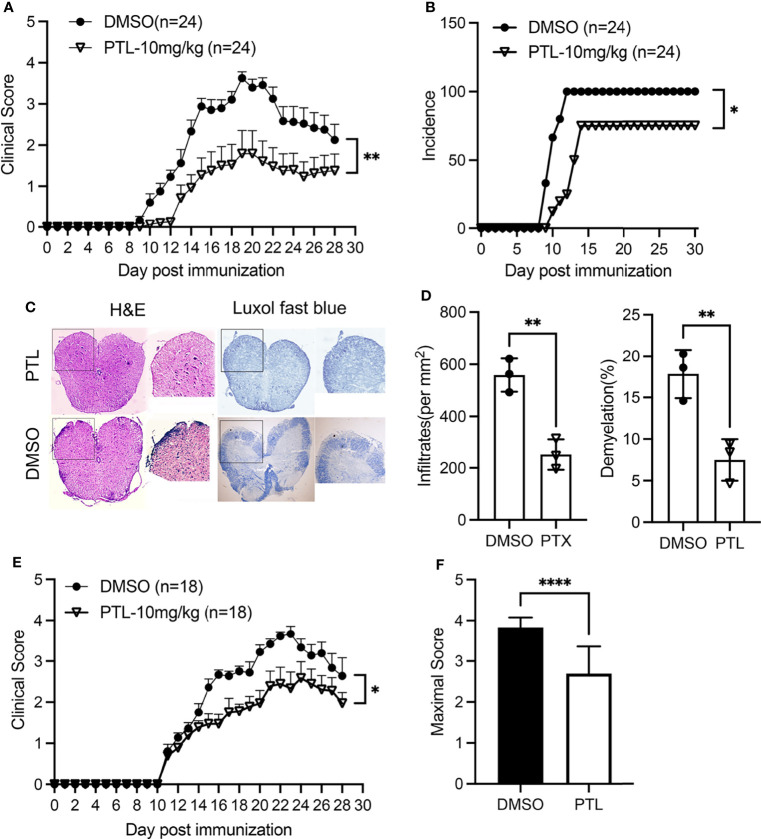
PTL ameliorated the clinical symptoms of EAE mouse. **(A)** Mean clinical scores of EAE mice treated with PTL (10 mg/kg) or DMSO (*n* = 24 mice/group). **(B)** Incidence of EAE in the DMSO group and PTL (10 mg/kg)-treated group (*n* = 24). **(C)** H&E staining and Luxol fast blue staining of spinal cords isolated from DMSO-treated mice and PTL-treated mice (*n* = 3 mice/group) on day 21 after immunization. **(D)** Quantification of spinal cord infiltrates and demyelination in the sections, presented as infiltrates per mm^2^ (left) and demyelination area relative to total analyzed area (right) of DMSO-treated mice and PTL-treated mice shown in **(D, E)** Mean clinical scores of EAE therapy with PTL (10 mg/kg) or DMSO (*n* = 18 mice/group). **(F)** Mean maximum clinical score of EAE mice treated with PTL (10 mg/kg) or DMSO. Data shown are representative of 3 independent experiments. The error bars shown were mean ± SD. *p < 0.05, **p < 0.01, ****p < 0.001 using unpaired Student’s *t*-test. Two-way analysis of variance (ANOVA) followed by Bonferroni’s post-hoc test, was applied for multiple comparisons. For EAE scoring, groups were compared using Mann**–**Whitney *U* test.

To explore the potential anti-inflammatory role of PTL in EAE, we treated mice with PTL (10 mg/kg) or vehicle control (DMSO) from day 1 post-immunization. Compared with the DMSO control group, PTL-treated mice showed lower clinical scores from day 10 to 28 after immunization (**Figure 1A**). Besides, treatment with PTL delayed the occurrence of EAE and decreased the EAE incidence (**Figures 1A, B**). In addition, treatment with PTL ameliorated inflammatory infiltration and demyelination in spinal cords, which were isolated from EAE mice on day 21 (**Figures 1C, D**). Furthermore, to investigate the therapy effects of PTL on EAE, EAE mice were treated with PTL or DMSO from day 11 (onset day) after immunization. Compared with the DMSO control group, PTL-treated mice showed obviously decreased clinical scores and mean maximal scores of EAE (**Figures 1E, F**). Collectively, these data suggested that PTL significantly ameliorated the clinical symptom of mice EAE.

### PTL-Treated Reduced Th1 and Th17 Response in CNS of EAE Mice

**Figure 2:**
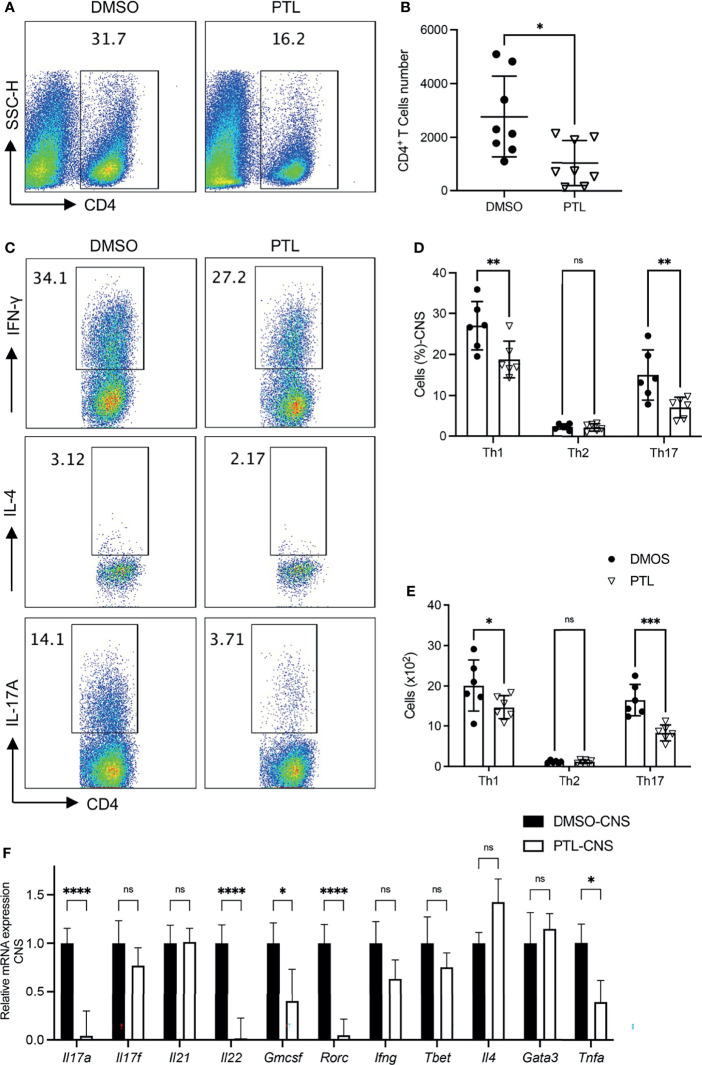
PTL treatment reduced Th1 and Th17 response in CNS of EAE mouse. Cells were isolated from the CNS of EAE mice treated with DMSO or PTL on day 21 after the immunizations and stimulated with PMA/ion plus golgi stop for 5 h **(A)** Percentage of CD4^+^ T cell and **(B)** absolute number of CD4^+^ T cell in CNS of EAE mice treated with PTL or DMSO (*n* = 8). **(C, D)** Percentage of Th1, Th2, and Th17 cells in CNS of two groups of EAE mice (*n* = 5). **(E)** Cell numbers of Th1, Th2, and Th17 cells in the CNS of two groups of EAE mice. **(F)** RT-PCR analysis of mRNA expression of Th1, Th2, and Th17 cell-related cytokines and transcription factors, in lymphocytes separated from the CNS of two groups of EAE mice (*n* = 3). Data shown are representative of 3 independent experiments. The error bars shown were the mean ± SD. *p < 0.05, **p < 0.01, ***p < 0.005, ****p < 0.001 using unpaired Student’s *t*-test. ns means “not significant”.

Pathogenic CD4^+^ T subsets in CNS play key roles during EAE progression. To investigate the function of PTL on pathogenic T cells in CNS, brain cells and spinal cord cells were harvested on day 21 of EAE mice. PTL treatment decreased the percentage (**Figure 2A**) and total cell number (**Figure 2B**) of CD4^+^ T cells compared with the DMSO control group. Furthermore, both the cell percentage and cell number of Th1 and Th17 cells were decreased by PTL treatment, while there is no significant change in Th2 cells between the PTL-treated group and the control group (**Figures 2C**–**E**). Consistently, mRNA expression of Th17-related inflammatory factors (*Il17a, Il17f, Il22*, and *Gmcsf*) and transcription factors (*Rorc*), and Th1 related inflammatory factors (*Ifng*) were downregulated, while transcription factors (*Tbet*), Th2-related inflammatory factors (*Il4*), and transcription factors (*Gata3*) were not affected by PTL treatment (**Figure 2F**). Taken together, our results suggested that PTL could reduce immune cell infiltration and suppress Th1 and Th17 responses, but not Th2 responses in the CNS during the peak phase of EAE.

### PTL Inhibited Th1 and Th17 Response in Peripheral Immune Organs of EAE Mice

**Figure 3:**
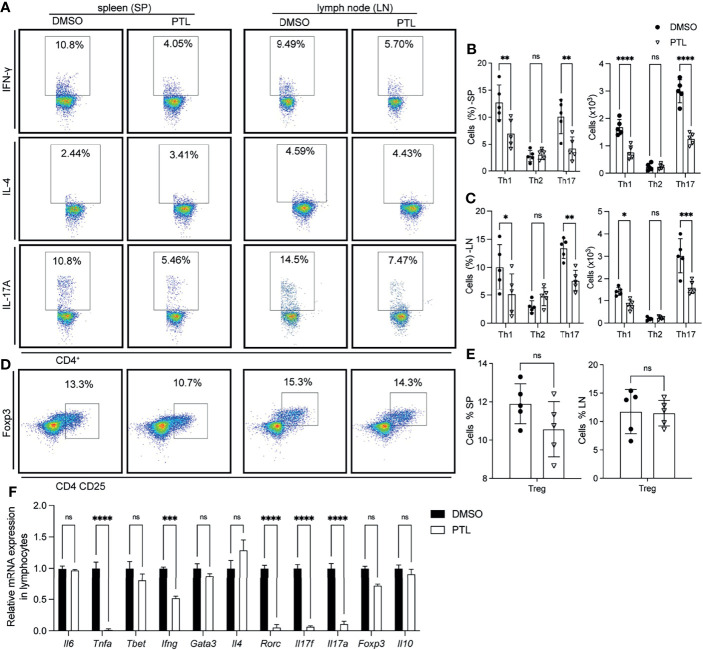
PTL inhibited Th1 and Th17 response in peripheral immune organs of EAE mice. Cells were isolated from the spleen (left) or lymph node (right) of EAE mice treated with DMSO or PTL (*n* = 6) on day 12 after the immunizations and stimulated with or without PMA/ion plus golgi stop for 5 h **(A)** Percentage of Th1 (represented by IFN-γ), Th2 (represented by IL-4), and Th17 (represented by IL-17A) in spleens and lymph node of two groups of EAE mice. **(B)** Statistical analysis of Th1, Th2, and Th17 cell numbers in spleens of two groups of EAE mice. **(C)** Statistical analysis of Th1, Th2, and Th17 cell numbers in lymph nodes of two groups of EAE mice. **(D)** Percentage of Tregs from spleen (left) and lymph node (right) of two groups of EAE mice. **(E)** Statistical analysis of Tregs number in spleen and lymph node. **(F)** RT-PCR analysis of mRNA expression of Th1, Th2, Th17, and Treg cell-related cytokines, transcription factors, and surface receptors in CD4^+^ T cells separated from spleen of two groups of EAE mice (*n* = 3) on day 13 after the immunizations. Data shown are representative of 3 independent experiments. The error bars shown were the mean ± SD. *p < 0.05, **p < 0.01, ***p < 0.005, ****p < 0.001 using unpaired Student’s *t*-test. ns means “not significant”.

To investigate whether the reduction of CD4^+^ T cell infiltration, especially Th1 and Th17 cells, in the CNS was caused by an abnormal activation in peripheral immune organs, we examined the Th1, Th2, Th17, and Treg cells in spleen and LN of PTL- and DMSO-treated EAE mice on day 11 after immunization. In both spleen and lymph node, PTL treatment inhibited the Th1 and Th17 cell response evidenced by decreasing the percentage and total number of IFN-γ- and IL-17A-positive Th cells. Nevertheless, the percentage and total number of Th2 cells were not significantly changed between the PTL-treated and DMSO control groups (**Figures 3A**–**C**). Both Th2 and Tregs cells are immune inhibitory cells and have protective roles in EAE development and progression. Since Th2 cell response was not affected by PTL, we further detected the Tregs in spleen and lymph node by labeled Tregs with CD4, CD25, and Foxp3 antibodies. PTL did not influence the percentage of Tregs in both spleen and lymph node (**Figures 3D, E**). To confirm these findings, RT-qPCR was performed to examine the mRNA expression of Th1-, Th2-, Th17-, and Treg cell-related cytokines in lymphocytes of PTL- and DMSO-treated EAE mice. As shown in **Figure 3F**, the expression of Th1 (*Ifng*)- and Th17 (*Il17a* and *Il17f*)-related cytokines significantly reduced after PTL treatment. In contrast, there were no significantly changes in Th2 (*Il4*)- and Treg (*Il10*)-related cytokines between two groups. These results suggested that PTL inhibited the response of Th1 and Th17 cells in peripheral immune organs during the onset of EAE.

### PTL Suppressed the Th1 and Th17 Cell Responses After MOG35–55 Re-Stimulation

**Figure 4:**
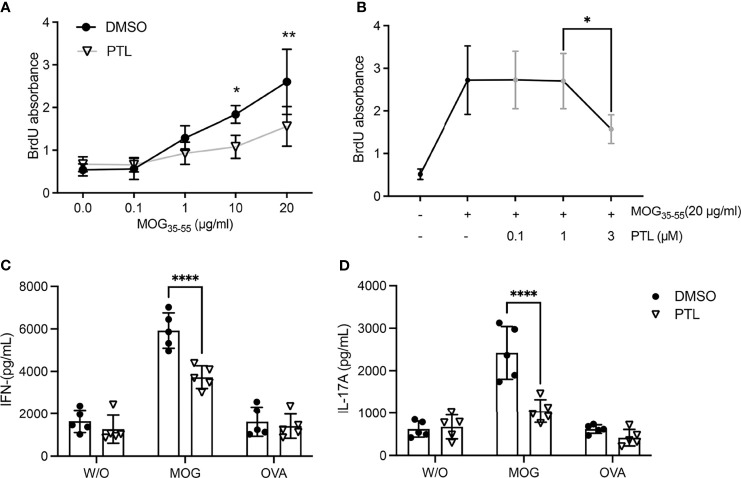
PTL modulates MOG_35–55_-reactive Th cell responses at day 21 after EAE induction. **(A)** Splenocytes were collected from spleen and lymph nodes of DMSO- and PTL-treated mice and stimulated by MOG_33-35_ for 48 h *in vitro*. MOG (0, 0.1, 1, 10, and 20 μg/ml) was added, and cell proliferation was measured by BrdU incorporation. **(B)** At day 21 after EAE induction, splenocytes were collected from spleen and lymph nodes and stimulated with or without MOG (20 μg/ml) for 48 h *in vitro*. PTL (0, 0.1, 1, and 3 μM) was added, and cell proliferation was detected by BrdU incorporation. At day 21 after EAE induction, lymphocytes were collected from spleen and lymph nodes of DMSO- and PTL-treated mice and cultured for 72 h without antigen (w/o) or with 20 µg/ml of the immunizing MOG or a non-relevant peptide (OVA); then, levels of interferon IFN-γ **(C)** and IL-17A **(D)** were measured by ELISA. The error bars shown were the mean ± SD. *p < 0.05, **p < 0.01, ****p < 0.001, using unpaired Student’s *t*-test. Two-way analysis of variance (ANOVA) followed by Bonferroni’s post-hoc test was applied for multiple comparisons.

To further explore whether the suppression function of PTL was related to MOG-specific T cells, the splenocytes from PTL- or DMSO-treated EAE mice were separated on day 21 after immunization and the proliferation was measured by BrdU stain after MOG_35-55_ peptide restimulation at the indicated concentrations. Results showed that MOG_35-55_ stimulated proliferation of splenocytes from DMSO-treated EAE mice in a dose-dependent manner, whereas the stimulatory effect was significantly lower on splenocytes from PTL-treated EAE mice (**Figure 4A**). Furthermore, PTL inhibited proliferation of splenocytes from EAE mice in the challenge of 20 μg/ml MOG_35–55_ in a dose-dependent manner (**Figure 4B**). To further explore the biological function of PTL on MOG-specific T-cell re-activation, we performed *ex vivo* antigen-specific recall assays and detected cytokine secretion in culture supernatant by ELISA. The results showed that MOG-specific T cells from PTL-treated mice produced less IFN-γ and IL-17A than those from DMSO treated mice (**Figures 4C, D**). In addition, OVA stimulation showed no obvious differences on IFN-γ and IL-17A levels between PTL- and DMSO-treated mice. These results suggested that PTL significantly inhibited the reactivation of MOG-specific Th1 and Th17 cell response.

### PTL Suppressed Th17 Differentiation

**Figure 5:**
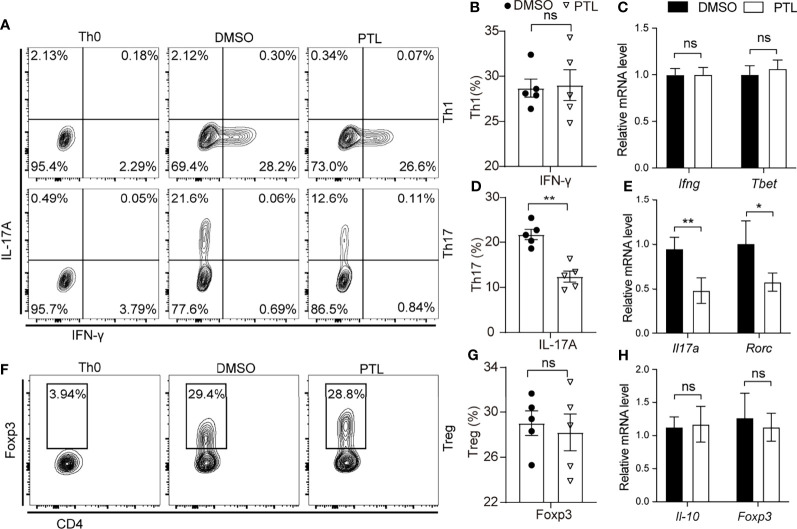
PTL suppressed Th17 differentiation. Naïve CD4^+^ T cells were sorted from spleen and lymph node and polarized with Th0, Th1, Th17, and Treg condition with or without PTL (3 μM) treatment for 4 days. **(A)** Flow cytometry was performed to detect the percentage of Th0, Th1, and Th17 of the DMSO group and PTL group. Statistical analysis of **(B)** Th1 and **(D)** Th17 in the PLT group and DMSO group. **(F)** Flow cytometry was performed to detect the percentage of Tregs in the DMSO group and PTL group. **(G)** Statistical analysis of Treg cell percentage in the DMSO and PTL-treated group. **(C, E, H)** The mRNA expression of Th1-, Th17-, and Treg-related cytokines and transcription factors was detected by RT-PCR after the differentiation. The error bars shown were the mean ± SD. *p < 0.05, **p < 0.01 using unpaired Student’s *t*-test. ns means “not significant”.

Since PTL could significantly inhibit Th1 and Th17 cell response in EAE mice, we further investigate the effect of PTL on the differentiation of naïve T cells. Naïve CD4^+^ T cells were sorted from spleen and LN and cultured with PTL under Th1, Th17, and Treg differentiation conditions for 4 days. Interestingly, PTL did not affect the differentiation of Th1 cell or Tregs, but significantly decreased the differentiation of Th17 (**Figures 5A**–**G**). In addition, RT-qPCR was used to detect the expression of representative cytokines, transcription factors, and Th cells. As shown in **Figures 5C, E, H**, PTL did not affect the expression of *Ifn-g*, *T-bet*, *Il-10*, and *Foxp3* significantly, but inhibited the expression of cytokine *Il-17a* and transcription factor *Rorc* in Th17 cells. These data further indicated that PTL could inhibit the differentiation of Th17 cells *in vitro*.

### PTL Inhibited Th17 Differentiation Through Suppressing the NF-κB/ROR-γt Pathway

**Figure 6:**
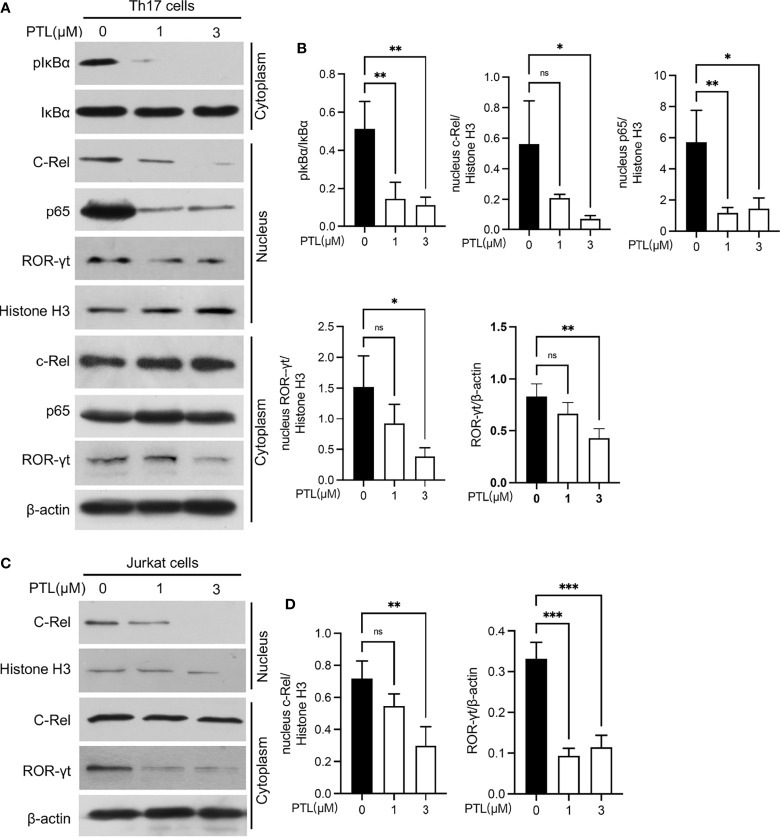
PTL inhibited Th17 differentiation through suppressing the NF-κB pathway. **(A)** Naïve CD4^+^ T cells were sorted from spleen and lymph node and polarized in Th17 condition with PTL (0,1 and 3 μM) for 48 h The expression of phosphorylated IκBα, IκBα, c-Rel, p65, and ROR-γt in the cytoplasm or nucleus was determined by Western blot. **(B)** The statistical results of Western blotting gray values from three independent repeated experiments were calculated. **(C)** Jurkat cells were treated with PTL (0, 1, and 3 μM) for 24 h. The expression of c-Rel and ROR-γt in cytoplasm or nucleus was detected by Western blot. **(D)** The statistical results of Western blotting gray values from three independent repeated experiments were calculated. The error bars shown were the mean ± SD. *p < 0.05, **p < 0.01, ***p < 0.005 using unpaired Student’s *t*-test. ns means “not significant”.

In order to systematically explore the effects of PTL on Th17 cells, we further studied the potential molecular mechanism of PTL on Th17 cell inhibition. Previous studies have shown that PTL could inhibit the NF-κB activation. Since the NF-κB pathway plays an important role in the processes of T-cell activation (36), we hypothesized that PTL may inhibit Th17 cell differentiation *via* regulating the NF-κB pathway. To verify this hypothesis, naïve CD4+ T cells were sorted from spleen and LN and cultured with PTL (0, 1, and 3 μM) under Th17 polarization conditions for 48 h. The protein expression of pIκBα in Th17 cells was significantly inhibited after PTL treatment. Consistently, p65 and c-Rel, when combined with IκBα in the cytoplasm, were significantly decreased in cell nucleus (**Figures 6A, B**). Based on the finding that the Rel-RORγt transcriptional axis plays critical roles in Th17 cell differentiation and response (31), we further investigated if PTL could suppress the ROR-γt expression under Th17 differentiation conditions. As expected, PTL significantly inhibited the ROR-γt signaling (**Figures 6A, B**). In addition, Jurkat cells were used to confirm the results we obtained in primary Th17 cells, and as shown in **Figure 6C**, PTL inhibited c-Rel and RORγt expression in Jurkat cells. Together, these results indicated that PTL could inhibit Th17 cell differentiation *via* suppressing NF-κB/ROR-γt.

## Discussion

PTL is extracted from the medicinal plant feverfew that has been applied for the treatment of intermittent fever for centuries (37). Current studies also showed that PTL has strong anti-tumor, anti-inflammatory, and anti-bacterial properties (16, 38). Recently, in a coronavirus disease 2019 (COVID-19) study, PTL served as a herbal candidate for COVID-19 clinical evaluation by reducing cytokines, including IL-1, IL-2, IL-8, and TNF-α, and inhibiting inflammatory signaling pathways (39). In this study, the effects of PTL on MS and EAE inflammation were investigated both *in vivo* and *in vitro*. Our data indicated the significantly protective impact of PTL on EAE, as evidenced by the reduction in clinical symptoms, incidence of EAE, inflammatory cell infiltration, and demyelination in CNS. We also found that PTL alleviated MS by inhibiting MOG-specific Th1 and Th17 proliferation, reactivation, and cytokine secretion. Furthermore, our data suggested that PTL inhibiting the differentiation of Th17 may be due to the suppression of the NF-κB/ROR-γt signaling pathway.

MS is a typical inflammatory demyelinating disease of the CNS. As a classical animal model of MS, EAE is widely used to investigate the pathogenesis and therapeutic methods of MS (40, 41). Since PTL has been reported to have anti-inflammatory properties, we detected whether PTL has an impact on inhibiting the auto-immune inflammation of EAE. EAE mice treated with PTL exhibited a lower clinical score and a lower incidence with PTL in a dose-dependent manner. Besides, PTL treatment could also inhibit inflammatory infiltration and demyelination in CNS, suggesting that PTL can act on MOG-induced autoimmune inflammatory response. In addition, PTL treatment after EAE induction (inject PTL around day 11 after immunization) showed a similar protective function of PTL on EAE, suggesting that PTL can not only prevent but also inhibit the progress of EAE.

T helper cells, mainly including Th1, Th2, Th17, and Treg cells, are important components of the adaptive immune system and are involved in autoimmune diseases (42, 43). Particularly, Th17 cells have been shown to take part in the pathogenesis of most autoimmune syndromes (2, 5, 44). In CNS of EAE mice, PTL significantly decreased CD4^+^ T cell percentage, especially Th1 and Th17, but not Th2 cells. In the meantime, PTL could also suppress Th1- and Th17-related cytokine levels, such as IL-17a, IL-17f, IL-22, GM-CSF, IFN-γ, and TNF-α in EAE mice CNS.

Infiltration of peripherally activated antigen-specific T cells into the CNS may trigger the onset of MS (45). Therefore, we investigated the influence of PTL on spleen and lymph nodes and showed that PTL can decrease the percentages of Th1 and Th17 cells, consistent with the Th1 and Th17 cell percentage in CNS.

Different from Th1 and Th17 cells, Th2 and Treg cells served as anti-inflammatory cells. As expected, PTL has no impact on Th2 and Treg cell percentages in both peripheral immune tissue and CNS. It was reported that PTL did not affect the absolute number and percentage of Treg cells in spleen of inflammatory bowel disease (IBD) mice (22), which is consistent with our data. Moreover, the mRNA expression of inflammatory and Th1-/Th17- related cytokines (TNF-α, IFN-γ, IL-17a, and IL-17f) was suppressed by PTL in lymphocytes from spleen and lymph node tissue of EAE mice. Together, PTL inhibited the response of Th1 and Th17 in peripheral immune organs, further suppressing the inflammatory infiltration of Th1 and Th17 into CNS *in vivo*.

Besides the *in vivo* study we discussed above, to further investigate PTL function *in vitro*, PTL- or DMSO-treated MOG-specific splenocyte T cells were re-stimulated with MOG_35-55_. PTL can effectively inhibit the proliferation of T cells that were re-stimulated by MOG_35-55_. Meanwhile, MOG-specific T cells from peripheral lymphatic organs of EAE mice were treated with MOG_35-55_ and PTL, showing that PTL inhibits MOG_35-55_-induced T-cell proliferation *in vitro* in a dose-dependent manner. In addition, PTL decreased Th1- and Th17-related cytokine secretion of antigen-specific T cells. These data support the conclusion of the previous study that PTL has immunosuppressive effects by regulating the activation of Th1 and Th17 *in vitro* (46). However, without *in vivo* study of PTL on an EAE mouse model, there is not enough evidence to draw the conclusion that PTL inhibits immune function. Together with the T-cell recall assays *ex vivo*, we demonstrated suppressive effects of PTL on autoimmune inflammation both *in vivo* and *in vitro*. Further study suggested that PTL could prevent activation and differentiation of Th17 *via* down-regulating the NF-κB/RORγt pathway.

Inhibition of T-cell proliferation and differentiation can be the reason that PTL prevents inflammatory reactivity. We detected that PTL inhibited Th17 cell differentiation *in vitro*. All inflammatory molecules secreted by Th17 were significantly inhibited by PTL treatment. However, Th1 cell differentiation was not affected by PTL. Combined with the results we obtained *in vivo* that PTL can inhibit the activation of Th1 in peripheral immune system and the expression of INF-γ, as well as other inflammatory factors, PTL may not directly inhibit Th1 cell function but there may be some other indirect mechanisms involved in the effects of PTL on Th1 cell response depending on the local immune microenvironment. As for Th2 and Treg, PTL did not influence either their proliferation or secretion *in vivo* and *in vitro*.

Studies have shown that in addition to helper T cells, other immune cells, such as dendritic cells, macrophage cells, CD8+ T cells, and B cells, also play an important role in the pathogenesis of EAE. PTL may improve the clinical symptoms of EAE by modulating the functions of other immune cells. *In vitro*, PTL could induce the secretion of IFN-γ and IL-13 from peripheral blood-derived CD8^+^ T cells (47). PTL could induce apoptosis in human B-lymphoma cell lines through NF-KB signaling (48). Studies also have demonstrated that PTL could mediate the maturation of dendritic cells (49) and macrophage cells (50). All these studies show that there may be other ways for PTL to treat EAE, which need to be further explored.

NF-κB signaling cascade is critical in the regulation of immune and inflammatory responses and has been linked to the pathogenesis of autoimmune demyelinating disease and other neurodegenerative disorders (51, 52). Mouse gene-targeting studies have revealed the important functions of each NF-κB subunit as regulators in various aspects of immunity. Studies have demonstrated that p105/50 deficiency can protect mice from EAE, which is associated with the failure of Th1 or Th2 effector cell differentiation from MOG-specific T cells (30). C-Rel regulates the expression of ROR-γt and is required for Th17 cell development, further explaining the ameliorated phenotype of c-Rel-deficient EAE mice (29). Because PTL can target several components of the NF-κB signaling pathway to inhibit the activation of NF-κB, we suspected if PTL may inhibit the activity of NF-κB to regulate the differentiation of Th cells and subsequently protect mice from EAE. Our study demonstrated that PTL can significantly inhibit the phosphorylation of IκBα and nuclear translocation of p65 and c-Rel, thereby regulating ROR-γt expression in mouse and human cells. These data suggested that the inhibitory effect of PTL on Th17 cell differentiation is through suppressing the NF-κB/ROR-γt pathway.

**Figure 7:**
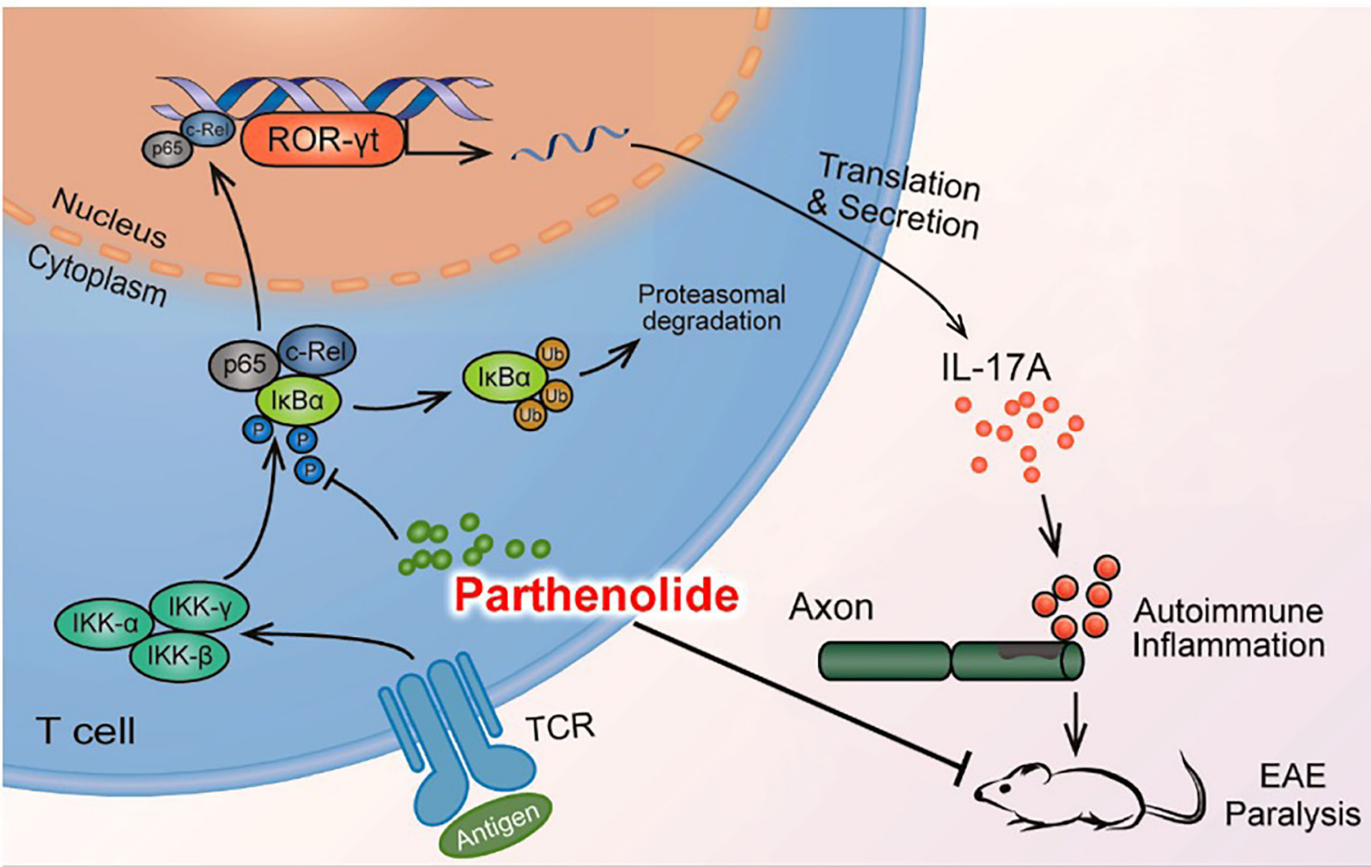
Mode pattern for the effect of PTL on the NF-κB/ROR-γt pathway.

In conclusion, our study provided evidence that PTL can inhibit Th17 cell differentiation by inhibiting the NF-κB/ROR-γt pathway, further resulting in the alleviation of EAE (**Figure 7**). These results suggested that PTL may become a potential therapeutic drug for autoimmune inflammatory diseases.

## Data Availability Statement

The original contributions presented in the study are included in the article/Supplementary Material. Further inquiries can be directed to the corresponding authors.

## Ethics Statement

The animal study was reviewed and approved by the Animal Care and Use Committee of the Tianjin Medical University Eye Hospital.

## Author Contributions

XZ, RZ, and ZZ designed the research and interpreted data. KZ, MZ, and ZZ performed experiments and analyzed data. ZZ wrote the manuscript. XZ and RZ reviewed the manuscript. All authors contributed to the article and approved the submitted version.

## Funding

This work was supported by the National Natural Science Foundation of China (82171042 and 81870651) and the Natural Science Foundation of Tianjin (20JCZDJC00100).

## Conflict of Interest

The authors declare that the research was conducted in the absence of any commercial or financial relationships that could be construed as a potential conflict of interest.

## Publisher’s Note

All claims expressed in this article are solely those of the authors and do not necessarily represent those of their affiliated organizations, or those of the publisher, the editors and the reviewers. Any product that may be evaluated in this article, or claim that may be made by its manufacturer, is not guaranteed or endorsed by the publisher.
